# Detection of pyridine derivatives by SABRE hyperpolarization at zero field

**DOI:** 10.1038/s42004-023-00928-z

**Published:** 2023-06-22

**Authors:** Piotr Put, Seyma Alcicek, Oksana Bondar, Łukasz Bodek, Simon Duckett, Szymon Pustelny

**Affiliations:** 1 Institute of Physics, Faculty of Physics, Astronomy and Applied Computer Science, Jagiellonian University in Kraków, Kraków, 30-348 Poland; 2 Goethe University Frankfurt, University Hospital, Institute of Neuroradiology, Frankfurt am Main, 60528 Germany; 3 Department of Chemistry, Taras Shevchenko National University of Kyiv, Kyiv, 01601 Ukraine; 4 Centre for Hyperpolarization in Magnetic Resonance (CHyM), University of York, Heslington, YO10 5NY UK

**Keywords:** Chemical physics, Physical chemistry, Solution-state NMR

## Abstract

Nuclear magnetic resonance (NMR) spectroscopy is a powerful analytical tool used in modern science and technology. Its novel incarnation, based on measurements of NMR signals without external magnetic fields, provides direct access to intramolecular interactions based on heteronuclear scalar *J*-coupling. The uniqueness of these interactions makes each zero-field NMR spectrum distinct and useful in chemical fingerprinting. However, the necessity of heteronuclear coupling often results in weak signals due to the low abundance of certain nuclei (e.g., ^15^N). Hyperpolarization of such compounds may solve the problem. In this work, we investigate molecules with natural isotopic abundance that are polarized using non-hydrogenative parahydrogen-induced polarization. We demonstrate that spectra of hyperpolarized naturally abundant pyridine derivatives can be observed and uniquely identified whether the same substituent is placed at a different position of the pyridine ring or different constituents are placed at the same position. To do so, we constructed an experimental system using a home-built nitrogen vapor condenser, which allows for consistent long-term measurements, crucial for identifying naturally abundant hyperpolarized molecules at a concentration level of ~1 mM. This opens avenues for future chemical detection of naturally abundant compounds using zero-field NMR.

## Abstract (web summary)

Zero- and ultralow-field (ZULF) nuclear magnetic resonance (NMR) has emerged as a complementary technique to conventional high-field (≳1 T) NMR, however, the ability to distinguish heteronuclear coupling remains challenging due to the low abundance of certain nuclei. Here, the authors apply non-hydrogenative parahydrogen-induced hyperpolarization to identify naturally abundant molecules, and demonstrate the detection of pyridine derivatives at a concentration level of ~1 mM.

## Introduction

Over the past decade, the zero- and ultralow-field (ZULF) nuclear magnetic resonance (NMR) technique has emerged as a complementary method to conventional high-field (≳1 T) NMR^1–4^. Its specific advantage is the ability to directly probe spin-spin interactions in zero field, where the Zeeman interaction, and hence the chemical shift, disappears, and the NMR signals are purely related with *J*-coupling. In turn, information-rich and chemically specific spectra (the so-called *J*-spectra)^5^ of narrow NMR lines (dipolar relaxation and chemical shift anisotropy are strongly suppressed)^6,7^ enabling high-resolution spectroscopy can be observed. Absence of strong field alleviates the necessity for cryogenic cooling, so that portable and inexpensive ZULF NMR spectrometers can be constructed, prophesizing a new era of NMR research^8–10^.

**Fig. 1:**
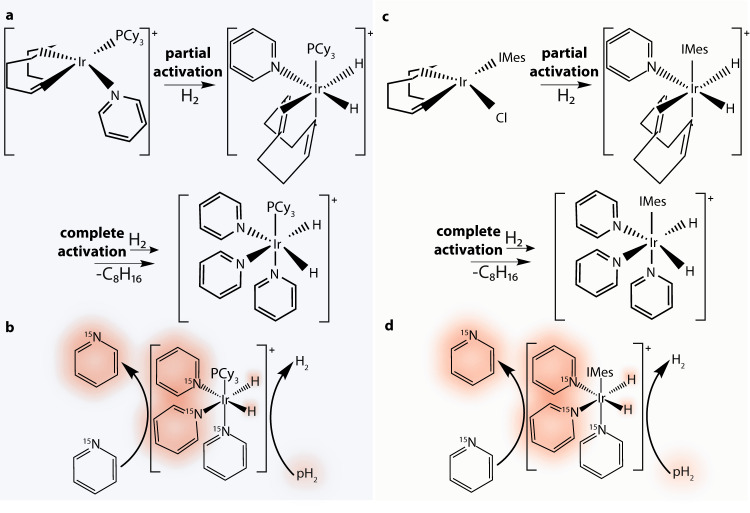
Chemical processes exploited in SABRE hyperpolarization. Formation of active SABRE hyperpolarization transfer catalyst for (**a**) Crabtree’s catalyst, [Ir(COD)(PCy_3_)(Py)]PF_6_ (COD = cyclooctadiene, Py = pyridine, PCy_3_ = tricyclohexylphosphine, PF_6_ = iridium(I) hexafluorophosphate), used for activation/reproducibility studies and (**c**) Ir-IMes catalyst, [IrCl(COD)(IMes)] (IMes = 1,3-bis(2,4,6-trimethylphenyl)imidazol-2-ylidene) used for investigations of pyridine derivatives. The details of the activation process can be found in Ref. ^48^. Chemical mechanism of SABRE process, resulting in hyperpolarization of molecules (highlighted in red) using (**b**) Crabtree’s catalyst and (**d**) Ir-IMes catalyst. The chemical mechanisms are shown for ^15^N-pyridine albeit analogue processes govern hyperpolarization of other pyridine derivatives studied in this work.

Since no magnetic field is present during the zero-field NMR measurements, samples need to be spin-polarized prior to the measurements. This can be achieved by remote thermal prepolarization with a permanent magnet located outside of the zero-field (detection) region^11^. This is a universal approach, providing, however, low sensitivity of the measurements. Alternatively, polarization of the sample can be achieved by application of chemically discerning hyperpolarization methods^12–15^ among which para-hydrogen-induced polarization (PHIP) is one of the most promising as it works well at ZULFs^16–19^. The resulting PHIP-signal enhancement can be achieved using hydrogenative PHIP^20–22^ or the so-called Signal Amplification by Reversible Exchange (SABRE)^23–26^, which is the method used in this work. SABRE employs a reversible interaction between an active iridium-based catalyst (formed from a precatalyst), a substrate (e.g., pyridine), and para-hydrogen (pH_2_) (Fig. 1). Due to this reversible interaction, a labile complex, containing protons from pH_2_ and the substrate, is formed. In such a complex, high polarization of pH_2_ protons is transferred to the molecule of interest through the *J*-coupling network of the catalyst (see Fig. 1b and d). As the active complex splits up, releasing the molecule into the solution, the structure of the molecule is unchanged while its spin polarization is high.

Observation of ZULF NMR spectra requires existence of heteronuclear *J*-coupling in the molecule of interest. Hence, low abundance of most spin-1/2 nuclei (e.g., ^13^C, ^15^N, ^29^Si) means that only a small fraction of molecules in the sample generates ZULF NMR signals. To address this problem, expensive isotopic labelling of molecules is needed^5^, with notable exceptions of molecules containing at least two of such spin-1/2 nuclei as ^1^H, ^19^F, and ^31^P of nearly 100% abundance^11,27^. The large polarization achieved through SABRE can alleviate the need for isotopically labelled compounds as the efficient sensitization of ^1^H, ^13^C, ^15^N, ^19^F, ^31^P, ^29^Si, etc., nuclei has proven possible with the use of SABRE technique^28–30^.

The SABRE technique is effective for catalytic hyperpolarization of such compounds as pyridine, which is a readily available molecular target now known to produce a high level of polarization^31,32^. While pyridine is toxic itself, it exhibits derivatives that are reflective of a wide range of pharmaceutical agents that show antimicrobial, antiviral, or anticancer properties^33^, while several promising biorelevant pyridine derivatives can be hyperpolarized^32,34–36^. The increased interest in the molecule and its derivatives also reflects their potential in magnetic resonance imaging (MRI) as contrast agents. For example, pyridine derivatives (nicotinic acid and nicotinamide) are B_3_ vitamers and have been hyperpolarized by SABRE to detect conventional NMR/MRI signals^37,38^. It should be noted that in this context pyridine was studied as a SABRE target not only in conventional NMR but also in ZULF NMR experiments^8,17^.

Although in zero field both chemical identification^5,9^ and application of various hyperpolarization methods^16,17^ have been demonstrated, until now they have not been studied in conjugation with each other. Without doubt, real-world applications of ZULF NMR will require both the ability to work with unaltered samples and to perform chemical identification^3^. This is the task that we undertake in this work by investigating a group of similar molecules –pyridine derivatives– in their natural isotopic abundance (containing 0.36% of ^15^N with spin-1/2) using ZULF NMR and SABRE hyperpolarization. We collect hyperpolarized zero-field spectra for natural abundant pyridine derivatives, not yet studied by ZULF NMR, and demonstrate that they can be distinguished by means of their zero-field spectra. This is done by studies of the pyridine derivatives containing the same substituent, at different ring positions, as well as various substituents at the same ring position. Among the different pyridine derivatives, we specifically target nicotinamide, which is the first biomolecule hyperpolarized with SABRE to be measured in zero field. To measure spectra with good signal-to-noise ratio, we develop an experimental system enabling reliable signal averaging. The system is based on a vapor condenser, which ensures a stable sample composition during long-term measurements. This experimental capability allowed for monitoring catalyst activation used in the SABRE process at zero magnetic field. Finally, our experimental results are supported with numerical simulations, which well reproduce all the spectra. This validates our theoretical approach and paves the way toward chemical identification of naturally abundant compounds using zero-field NMR.

## Results and discussion

### In-situ catalyst activation and long-term signal monitoring with ZULF NMR

**Fig. 2:**
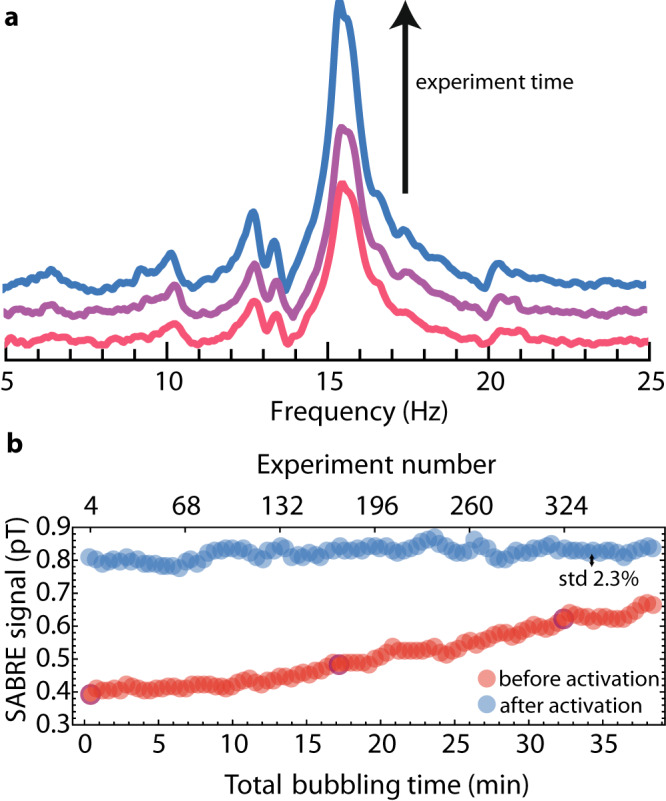
Time dependence of the zero-field NMR signal of SABRE-polarized pyridine. **a** Zero-field spectrum of SABRE-polarized ^15^N-pyridine during prolonged para-hydrogen bubbling. The NMR signal amplitude increases as the SABRE catalyst activates (the spectra were shifted in the vertical direction for better visibility). **b** Signal intensity (red points) as a function of time the precatalyst was exposed to pH_2_ (the nonzero initial response indicates the sample was partially activated at measurement onset). Darker, shaded data points correspond to the NMR spectra shown in part (**a**). Analogous signal-intensity profile (blue points) is shown for a sample that was fully activated prior to the measurements. The signal high reproducibility, with 2.3% variation in signal intensity over hundreds of transients, is demonstrated. To facilitate these measurements, a 70 mM solution of labelled [^15^N]-pyridine was used to boost the zero-field signal.

A zero-field NMR signal can be used to directly measure the efficiency of polarization transfer during repeated SABRE experiments. Figure 2a presents the ZULF NMR spectrum of pyridine measured at different times after the experiment initiation. As shown, the amplitude of the signal increases with the length of the experiment. Moreover, because the area under the ZULF NMR spectrum (signal intensity) can be used as a quantitative measure of the efficiency of the polarization transfer, Fig. 2b shows the ZULF NMR spectrum of pyridine integrated between 5 and 25 Hz as a function of the time of bubbling pH_2_ through the solution. For this study, each transient was measured for 6 s, and each resulting data point was averaged over four successive transients. The data presented in Fig. 2b (red points) shows the initial growth of signal intensity over time indicating an improvement in the polarization efficiency. Interestingly, the signal starts at a nonzero value, which indicates operation with a partially activated sample. The results of a similar experiment, starting with a fully activated catalyst, are overlayed in Fig. 2b (blue points). The activation was achieved by purging the sample with a large volume of pH_2_ (10 min purge with a flow of 300 sccm) before starting the measurement. This, combined with the condenser keeping the sample composition stable over time (see discussion below), results in the detection of hundreds of stable (~2% standard deviation) zero-field NMR signal transients with signal amplitude twice that observed during the first measurement (red points Fig. 2b). As catalyst activation provides stronger zero-field signals, it was used in the subsequent studies of naturally abundant pyridine derivatives.

The data presented in Fig. 2b, as well as spectra of natural abundant molecules presented in the next section, are the result of long-term experiments (tens of minutes of total pH_2_ bubbling). The constant flow of dry gas can result in the evaporation of an NMR sample, causing changes in spectra and polarization efficiency^39^. Additionally, when an NMR sample reflects a high concentration of analyte, the evaporation of solvent will cause precipitation. In this case, precipitation can lead to capillary obstruction, resulting in interrupted pH_2_ bubbling during the SABRE experiment. In our work, a vapor condenser, using a cold nitrogen vapor coolant, was introduced to enable long-term measurement (see Methods sections and Fig. 4). The cold nitrogen vapor in the condenser keeps the top of the NMR tube at a lower controllable temperature, allowing the solvent vapor to recondense and return to the bottom of the tube as liquid (the bottom of the NMR is heated up to ~40 °C, see Methods section). When the condenser temperature is maintained at about 0 °C, a sample of a volume of 0.5 ml can be used to record NMR signals for over 7.5 h (allowing for up to 10 times longer measurement than presented in the following section). Furthermore, increasing the flow of the liquid nitrogen vapor through the condenser would make even longer measurements possible. As shown in Fig. 2b, the use of condenser results in the robustness of the SABRE protocol (same amplitude over time after activation), delivering similar observations over at least 35 min, which is an important observation given the known dependence on the SABRE response on concentration. An alternative method to prolong SABRE experiments, where the gas is presaturated with methanol vapor, has been described in the literature^39^. Despite the effectiveness of this technique it requires use of extra solvent volume, while controlling the condenser temperature could be viewed as an advantage of the method used in our study.

While the initial investigations on the efficiency of polarization transfer in SABRE were performed with Crabtree’s catalyst, in order to enable the detection of much weaker NMR signals from naturally abundant compounds (presented in the following section), a change was made to the experimental protocol. Commercially available Crabtree’s catalyst was replaced with [IrCl(COD)(IMes)] precatalyst, which is known to yield higher hyperpolarization levels for pyridine and its derivatives (activation process and hyperpolarization mechanism are shown in Fig. 1c, d)^40^.

### Natural abundance SABRE at zero field

**Fig. 3:**
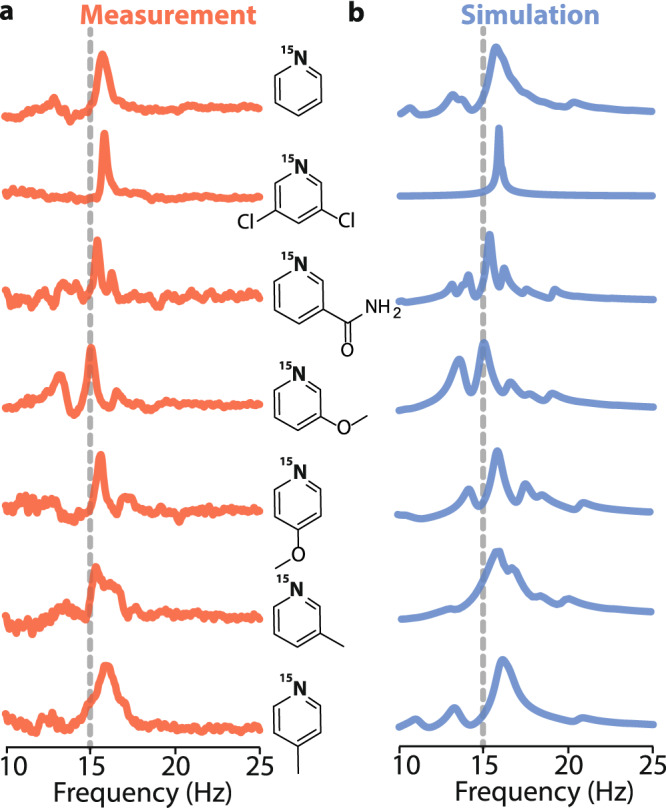
Zero-field NMR spectra of the SABRE-polarized molecules at natural isotopic abundance. **a** NMR spectra of a range of pyridine derivatives hyperpolarized and measured in zero magnetic field. Those are in order from the top: ^15^N-pyridine, ^15^N-3,5-dichloropyridine, ^15^N-nicotinamide, ^15^N-3-methoxypyridine, ^15^N-4-methoxypyridine, ^15^N-3-methylpyridine, ^15^N-4-methylpyridine. **b** Numerical simulations of the zero-field spectra of the corresponding pyridine derivatives (see Methods for simulation details). The dashed line indicates a position of zero-field line corresponding to the strongest *J*-coupling between ^15^N and two neighbouring protons, present in all of the studied molecules.

Figure 3 presents zero-field NMR spectra of hyperpolarized pyridine derivatives: 3,5-dichloropyridine, 3-methoxypyridine, 4-methoxypyridine, 3-methylpyridine, 4-methylpyridine, and nicotinamide. Due to the need for a heteronuclear coupling, the spectra exclusively correspond to ^15^N-pyridine, ^15^N-3,5-dichloropyridine, ^15^N-3-methoxypyridine, ^15^N-4-methoxypyridine, ^15^N-3-methylpyridine, ^15^N-4-methylpyridine, and ^15^N-nicotinamide, which are present only at a level of 0.36% due to the ^15^N abundance. In these molecules, the strongest *J*-coupling, ^2^*J*_NH_, arises between ^15^N and the two ortho-protons of the pyridine ring. Thereby, the main peaks observed in the spectra of the molecules appear at 3/2  ^2^*J*_NH_, which corresponded to ≈ 15 Hz (indicated by the dashed line in Fig. 3)^41^. It is clear from these data that, when these dominant *J*-couplings are similar, the weaker (at least three-bond) hetero- (^1^H-^15^N) and homonuclear *J*-couplings (^1^H-^1^H) lead to the modification of the spectra. These modifications are responsible for uniqueness of zero-field NMR spectra and pave avenues for chemical analysis with the technique. It should be noted that analytical calculations of zero-field spectra are only possible in the simplest spin topologies. In a more involved spin system, yet, when the strength of the couplings forms a clear hierarchy, one may implement a perturbative approach to determine the structure of zero-field spectra^42^. Such calculations can provide more complex, yet analytical formula for shift and splitting of the NMR lines. However, in molecules without such a clear hierarchy, one may not be able to accurately calculate the spectra using perturbation theory approaches. In such a case, the simulations of the spectra may be performed based on numerical diagonalization of the spin Hamiltonian. Although this technique offers the most precise calculation and reproduction of the experimental spectrum, it does not provide simple means for the interpretation of the observed spectra.

We start with the discussion of the ^15^N-3,5-dichloropyridine spectrum (second spectrum from the top in Fig. 3). In this spectrum, a single line at 15.7 Hz is observed. The line is narrower than the corresponding line in the ^15^N-pyridine spectrum (top spectrum in Fig. 3) visible at 15.4 Hz. This narrowing is a result of a substitution of two hydrogen atoms by self-decoupled chlorine atoms^27^, which reduces the number of weak *J*_NH_-interactions and limits splitting of the main peak (the splitting is smaller than the resonance width, so its absence manifests as the line narrowing). A similar argument can be made when discussing the ^15^N-nicotinamide spectrum (third from the top in Fig. 3), where the amide substituent does not have a coupling to a single proton.

Another result of the substitution of hydrogens is a violation of the chemical symmetry of the two ortho-protons, as in ^15^N-nicotinamide, ^15^N-3-methoxypyridine, and ^15^N-3-methylpyridine. The change influences the bonding in these molecules, which leads to the dependence of the *J*-coupling constant on the position in the molecule. This leads to the appearance of several additional peaks of relatively high amplitude observed in the zero-field spectra around the main line. It should be noted that the lower signal-to-noise ratio seen in the ^15^N-nicotinamide spectrum compared to other spectra is a consequence of the use of a lower substrate concentration due to its low solubility in methanol. In our study, this issue was partially addressed by using dimethyl sulfoxide (DMSO) as a co-solvent. This particular result demonstrates the use of the SABRE technique to polarize and detect a spectrum of the biomolecule, B_3_ vitaminer, under zero-field conditions for the first time ever. In a way, this result hints at the possibility of using hyperpolarized nicotinamide for ultralow-field imaging^37,43^.

When methyl is a substrate in a pyridine-based molecule, additional (weak) *J*-couplings arise, forming a more complex spin-spin network. This additional interaction leads to splitting of the zero-field NMR lines, and it manifests as broadening of the main peak in both ^15^N-3-methylpyridine and ^15^N-4-methylpyridine spectra (Fig. 3). Once again this broadening originates from the inability to resolve splitting of the lines (in simulations, the influence of methyl protons is implemented using a simple line broadening function). On the other hand, narrower lines are observed in the spectra of ^15^N-3-methoxypyridine and ^15^N-4-methoxypyridine even though a methoxy group also contains three ^1^H nuclei. The reason for this is the much weaker (longer-range) *J*-couplings between ^1^H nuclei in the methoxy group and ^15^N compared to those in the methyl group. On the other hand, there was no spectral peak arising from ^13^C-isotopomers at their natural abundance. This finding might be explained by a reduction in the efficiency of hyperpolarization transfer to ^13^C in the presence of quadrupolar nuclei (i.e., ^14^N)^44^ and the complex spin system of ^13^C-^15^N isotopomers resulting in numerous split lines with low peak amplitudes^17^.

The presented experimental results are supported by the results of numerical simulations of ZULF NMR spectra (Fig. 3b). Such simulations are performed with the *J*-coupling constants taken from the literature. For more details on the simulation parameters and coupling networks see Supplementary Figure 2 and Supplementary Table 1,2,3,4, while the simulation script is included as Supplementary Data 2. Theoretical model reflects only free ligands (pyridine derivatives) in the solution as T_2_ of the bound compounds is short making them not detectable with zero-field NMR. All the simulated spectra match experimental results in both the positions and relative amplitudes of the NMR lines (raw data can be found in Supplementary Data 1). The discussion presented in this section shows that each pyridine derivative has either a different spin topology or the spin network is affected by different values of the *J*-coupling constants between different atoms. Thereby a unique zero-field NMR spectrum is associated with each molecule, demonstrating the ability of application of zero-field NMR for chemical identification. It should be stressed that such identification is possible with samples containing 1–2 M pyridine derivatives at natural abundance of ^15^N (corresponding to 3.6–7.2 mM of NMR active molecules) by measuring less than 200 transients. This, combined with the robust of the experimental protocol described above, suggests the possibility of SABRE-enhanced ZULF NMR measurements with naturally abundant ^15^N-compounds at millimolar concentration levels.

## Conclusions

In this work, we investigated SABRE-polarization at zero-magnetic field for a range of molecules: pyridine derivatives at their natural isotopic abundance. Using zero-field NMR, we analyzed the molecular spectral patterns and demonstrated their uniqueness with respect to both the substituent and its position. Such identification was performed for the first time using hyperpolarized samples at natural isotopic abundance. Along with the measurements, the experimental setup was constructed, allowing long-term in-situ SABRE experiments performed under zero-field conditions. It enabled studies of the activation of the SABRE catalyst for optimal zero-field hyperpolarization protocol, as well as long-term (many averages) measurements, allowing for the study of compounds at natural isotopic abundance of ^15^N. A particularly promising extension of the current study is the development of in-situ SABRE-RELAY hyperpolarization for ZULF NMR^45^. This would expand the range of molecules that could be investigated without the need for expensive labeling. With this we improved the applicability of zero-field NMR spectroscopy as a specific, sensitive, compact, and inexpensive method for chemical and biochemical analysis, paving the way for further applications of the technique in biochemical studies and fundamental-physics investigations (e.g., spin-gravity coupling searches).

## Methods

### Sample preparation

For monitoring the SABRE-catalyst activation and long-term signal stability check, a sample with 70 mM of ^15^N-pyridine (CAS#34322-45-7) and 2 mM of Crabtrees’s catalyst, [Ir(COD)(PCy_3_)(Py)]PF_6_, (CAS# 64536-78-3) was prepared in 300 μL of methanol.

For the study of naturally abundant ^15^N-pyridine derivatives, chemicals: pyridine (CAS# 110-86-1), 3,5-dichloropyridine (CAS# 2457-47-8), nicotinamide (CAS# 98-92-0), 3-methoxypyridine (CAS# 7295-76-3), 4-methoxypyridine (CAS# 620-08-6), 3-methylpyridine (CAS# 108-99-6), 4-methylpyridine (CAS# 108-89-4), and methanol (CAS# 67-56-1) were purchased from Sigma-Aldrich and used without further purification. The NMR samples consisted of a 25 mM of air-stable SABRE precatalyst, [IrCl(COD)(IMes)], mixed with 2 M solution of the corresponding pyridine derivatives (except for nicotinamide) were dissolved in 500 μL of methanol. Due to the low solubility of nicotinamide in methanol, a 1 M nicotinamide solution was prepared by addition of 20 μL of DMSO and 480 μL of methanol to 25 mM of the precatalyst.

Para-hydrogen (44% of pH_2_) was produced by passing pure hydrogen gas through a cold tubing (77 K) filled with an iron (III) oxide catalyst (a home-built para-hydrogen generator). Produced pH_2_ filled an aluminium cylinder, which was then connected to a bubbling system to expose the NMR sample to the gas. Initially, the precatalyst was activated in Earth’s field by bubbling pH_2_ through the samples for a few minutes. The change in the sample colour (the sample becoming transparent) was accepted as an indication of catalyst activation. The sample was then placed into the zero-field NMR spectrometer. The SABRE experiments were carried out by bubbling pH_2_ through sample solutions located in the zero-field region for 6–15 s at a flow rate of ~80 sccm and pressure of 5 bar. After termination of the bubbling, a magnetic-field pulse (*π*/2 rotation for ^1^H) was applied and the acquisition of the signal started. For details of the experimental sequence see Supplementary Note 1 and Supplementary Fig. 1.

### Zero-field NMR spectrometer

**Fig. 4:**
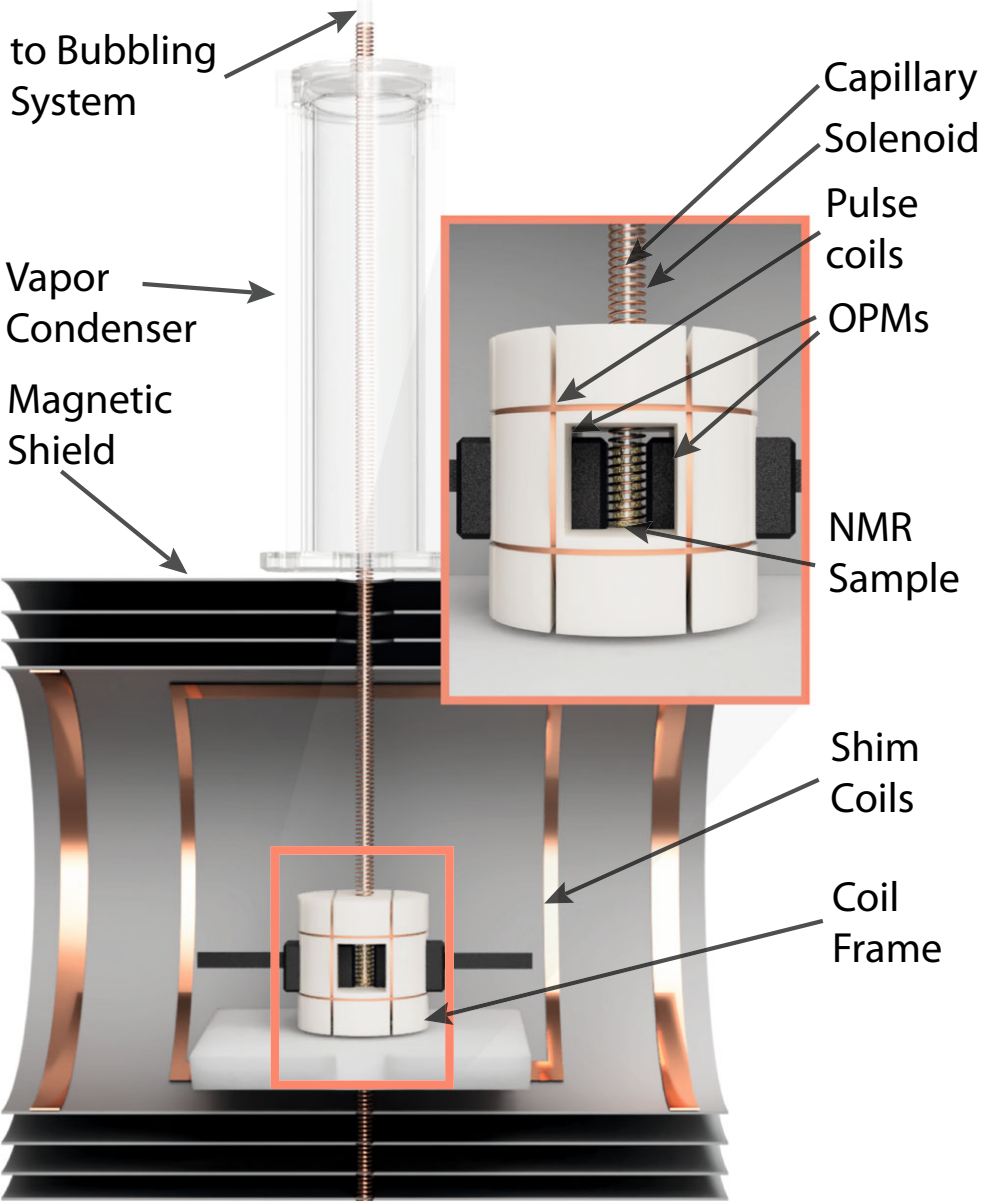
The key components of the ZULF NMR spectrometer used for the measurements. A liquid sample resides inside a long, 5 mm in diameter NMR tube placed inside a magnetic shield, providing shielding from external magnetic field and zero-field conditions. The bottom of the tube is positioned inside a coil frame, right next to two OPMs. The frame supports 3 pairs of orthogonal coils, which are used to generate DC magnetic-field pulses. The magnetic field at the position of the sample is zero-out using a set of shim coils connected to precision current source. The NMR tube is placed inside the solenoid (not used here) tube piercing the whole length of the shield. The top of the NMR tube is connected to the rest of bubbling system (for more details see Supplementary Fig. 1) and provides an inlet for pH_2_ gas through a thin capillary. The top of the tube is placed inside a chamber cooled with liquid nitrogen vapor, to recondense the sample solvent carried out with the gas.

The key components of the ZULF NMR spectrometer (prototype by DM Technologies) used in this work are shown in Fig. 4. Two commercial, ready-to-use Optically-Pumped Magnetometers (OPMs) (QuSpin QZFM) were used for the detection of weak, slowly-oscillating magnetic field, originating from NMR samples. Although the magnetometers could simultaneously detect two components of the field, only one direction per sensor was used. This choice was motivated by a fixed direction of the oscillations of samples’ magnetization at the zero field. The magnetometers were placed in a 3D-printed coil frame and held in the closest position to the NMR sample contained in a 5 mm glass tube. To attenuate external magnetic fields a compact, three-layer mu-metal shield (Twinleaf MS1F) with an additional ferrite innermost layer was used. The magnetometer and the sample during the detection were enclosed inside the shield. An absolute zero field (<0.1 nT) was achieved using a set of magnetic-field coils located inside the shielding. At the same time, additional coils, mounted on the smaller frame closer to the sample, were used to apply DC magnetic-field pulses. The top of the NMR tube was connected to a bubbling system, which used a pair of solenoid valves to control the flow of para-hydrogen through a small capillary submerged in the NMR sample (a more detailed description of the bubbling system and the typical experimental sequence can be found in Supplementary Note 1 and Supplementary Fig. 1). The top of the tube was also enclosed in the vapor condenser, a 3D-printed chamber, that was cooled with liquid nitrogen vapor. This minimized evaporation of the sample. Magnetometer signals were acquired using a 16-bit DAQ card (NI USB-6002), which was also used to generate the experimental sequence, controlling solenoid valves for bubbling, magnetic-field pulses, etc. The experimental sequence was pre-programmed with the use of LabView software that run on a PC. The computer was also used for data acquisition.

### Data postprocessing and spectra simulation

Magnetometer data (2000 Sa/s) acquired from two sensors were first de-based using a polynomial fit that subtracted slow drift of the magnetometer signal. Due to the finite bandwidth of the OPMs, the initial 35 ms of the signals were cut off, as the magnetometer response was saturated by the magnetic-field pulse used to generate the NMR signal. The cut points were reconstructed using autoregressive backprediction. Additional exponential apodization was applied to account for non-optimal signal acquisition time (10 s for all experiments) and hence improve the signal-to-noise ratio. Time signals from both OPMs were then Fourier transformed, phase corrected, and combined to produce a final zero-field NMR spectrum. Data post-processing was performed using Wolfram Mathematica.

The experimental spectra are accompanied by the simulation of zero-field NMR spectra based on the numerical diagonalization of the density matrix in the basis of the zero-field Hamiltonian^1^ and subsequent determination of the magnetization along the sensitive axis of the OPM. Calculations were performed using the high-performance spin simulation library Spintrum^46,47^, while the form of the density matrix following a magnetic-field pulse was assumed to be proportional to the nuclear polarization created along the sensitive direction of the sensor. This provided an adequate match to the experimental results. The relevant *J*-coupling constants used in the spectra simulations were taken from the literature and are summarized in Supplementary Tables 1, 2, 3, 4. In addition, line broadening was applied using a decaying single-exponential apodization function to simulated spectra based on experimental results.

## Supplementary information

Supplemental Information

Description of Additional Supplementary Files

Supplementary Data 1

Supplementary Data 2

## Data Availability

All raw experimental and simulated NMR spectra are available in Supplementary Data 1.

## Code availability

Zero-field NMR spectra simulation code based on the Spintrum library^47^ is included in Supplementary Data 2.

## References

[1] Ledbetter M (2009). Optical detection of NMR J-spectra at zero magnetic field. J. Magn. Reson..

[2] Blanchard JW, Budker D (2016). Zero- to ultralow-field NMR. eMagRes.

[3] Blanchard JW, Budker D, Trabesinger A (2021). Lower than low: perspectives on zero- to ultralow-field nuclear magnetic resonance. J. Magn Reson..

[4] Weitekamp DP, Bielecki A, Zax D, Zilm K, Pines A (1983). Zero-field nuclear magnetic resonance. Phys. Rev. Lett..

[5] Blanchard JW (2013). High-resolution Zero-Field NMR J-spectroscopy of aromatic compounds. J. Am. Chem. Soc..

[6] Emondts M (2014). Long-lived heteronuclear spin-singlet states in liquids at a zero magnetic field. Phys. Rev. Lett..

[7] Burueva DB (2020). Chemical reaction monitoring using zero-field nuclear magnetic resonance enables study of heterogeneous samples in metal containers. Angewandte Chemie International Edition.

[8] Blanchard JW, Wu T, Eills J, Hu Y, Budker D (2020). Zero-to ultralow-field nuclear magnetic resonance J-spectroscopy with commercial atomic magnetometers. J. Magn. Reson..

[9] Put P (2021). Zero-to ultralow-field NMR spectroscopy of small biomolecules. Anal. Chem..

[10] Alcicek S, Put P, Barskiy D, Kontul V, Pustelny S (2021). Zero-field NMR of Urea: Spin-topology engineering by chemical exchange. J. Phys. Chem. Lett..

[11] Tayler MC, Sjolander TF, Pines A, Budker D (2016). Nuclear magnetic resonance at millitesla fields using a zero-field spectrometer. J. Magn. Reson..

[12] Nikolaou P, Goodson BM, Chekmenev EY (2015). NMR hyperpolarization techniques for biomedicine. Chem. Eur. J..

[13] Barskiy DA (2017). NMR hyperpolarization techniques of gases. Chem. Eur. J..

[14] Kovtunov K (2018). Hyperpolarized NMR spectroscopy: d-DNP, PHIP, and SABRE techniques. Chem. Asian J..

[15] Eills J (2023). Spin hyperpolarization in modern magnetic resonance. Chem. Rev..

[16] Theis T (2011). Parahydrogen-enhanced zero-field nuclear magnetic resonance. Nat. Phys..

[17] Theis T (2012). Zero-field NMR enhanced by parahydrogen in reversible exchange. J. Am. Chem. Soc..

[18] Eills J (2019). Polarization transfer via field sweeping in parahydrogen-enhanced nuclear magnetic resonance. J. Chem. Phys..

[19] Eills, J. et al. Metabolic reactions studied by zero- and low-field nuclear magnetic resonance. *arXiv.* Preprint. 2205.12380 (2022).

[20] Bowers CR, Weitekamp DP (1986). Transformation of symmetrization order to nuclear-spin magnetization by chemical reaction and nuclear magnetic resonance. Phys. Rev. Lett..

[21] Bowers CR, Weitekamp DP (1987). Parahydrogen and synthesis allow dramatically enhanced nuclear alignment. J. Am. Chem. Soc..

[22] Green RA (2012). The theory and practice of hyperpolarization in magnetic resonance using parahydrogen. Prog. Nucl. Magn. Reson. Spectrosc..

[23] Adams RW (2009). Reversible interactions with para-hydrogen enhance NMR sensitivity by polarization transfer. Science.

[24] Adams RW, Duckett SB, Green RA, Williamson DC, Green GGR (2009). A theoretical basis for spontaneous polarization transfer in non-hydrogenative parahydrogen-induced polarization. J. Chem. Phys..

[25] Atkinson KD (2009). Spontaneous transfer of parahydrogen derived spin order to pyridine at low magnetic field. J. Am. Chem. Soc..

[26] Rayner PJ (2017). Delivering strong ^1^H nuclear hyperpolarization levels and long magnetic lifetimes through signal amplification by reversible exchange. Proc. Natl Acad. Sci..

[27] Alcicek S, Put P, Kontul V, Pustelny S (2021). Zero-field NMR J-spectroscopy of organophosphorus compounds. J. Phys. Chem. Lett..

[28] Roy SS, Appleby KM, Fear EJ, Duckett SB (2018). SABRE-Relay: a versatile route to hyperpolarization. J. Phys. Chem. Lett..

[29] Rayner PJ, Richardson PM, Duckett SB (2020). The detection and reactivity of silanols and silanes using hyperpolarized ^29^Si nuclear magnetic resonance. Angewandte Chemie International Edition.

[30] TomHon P (2021). Temperature cycling enables efficient ^13^C SABRE-SHEATH hyperpolarization and imaging of [1-^13^C]-pyruvate. J. Am. Chem. Soc..

[31] Theis T (2015). Microtesla SABRE enables 10% nitrogen-15 nuclear spin polarization. J. Am. Chem. Soc..

[32] Fekete M, Ahwal F, Duckett SB (2020). Remarkable levels of ^15^N polarization delivered through SABRE into unlabeled pyridine, pyrazine, or metronidazole enable single scan NMR quantification at the mM level. J. Phys. Chem. B.

[33] Khan E (2021). Pyridine derivatives as biologically active precursors; organics and selected coordination complexes. ChemistrySelect.

[34] Fekete M, Rayner PJ, Green GGR, Duckett SB (2017). Harnessing polarisation transfer to indazole and imidazole through signal amplification by reversible exchange to improve their NMR detectability. Magn. Reson. Chem..

[35] Durst M (2016). *α*-trideuteromethyl[^15^N]glutamine: a long-lived hyperpolarized perfusion marker. Magn. Reson. Med..

[36] Jagtap AP, Kaltschnee L, Glöggler S (2019). Hyperpolarization of ^15^N-pyridinium and ^15^N-aniline derivatives by using parahydrogen: new opportunities to store nuclear spin polarization in aqueous media. Chem. Sci..

[37] Rovedo P (2016). Molecular MRI in the Earth’s magnetic field using continuous hyperpolarization of a biomolecule in water. J. Phys. Chem. B.

[38] Olaru AM, Burns MJ, Green GGR, Duckett SB (2017). SABRE hyperpolarisation of vitamin B_3_ as a function of pH. Chem. Sci..

[39] Blanchard JW (2021). Towards large-scale steady-state enhanced nuclear magnetization with in situ detection. Magn. Reson. Chem..

[40] Cowley MJ (2011). Iridium n-heterocyclic carbene complexes as efficient catalysts for magnetization transfer from para-hydrogen. J. Am. Chem. Soc..

[41] Theis T (2013). Chemical analysis using J-coupling multiplets in zero-field NMR. Chem. Phys. Lett..

[42] Butler MC (2013). Multiplets at zero magnetic field: the geometry of zero-field NMR. J. Chem. Phys..

[43] Linnik I, Rayner P, Stow R, Duckett S, Cheetham G (2019). Pharmacokinetics of the SABRE agent 4,6-d2-nicotinamide and also nicotinamide in rats following oral and intravenous administration. Eur. J. Pharm. Sci..

[44] Barskiy DA (2017). The absence of quadrupolar nuclei facilitates efficient 13c hyperpolarization via reversible exchange with parahydrogen. ChemPhysChem.

[45] Dyke ETV (2022). Relayed hyperpolarization for zero-field nuclear magnetic resonance. Sci. Adv..

[46] Wilzewski A, Afach S, Blanchard JW, Budker D (2017). Method for measurement of spin-spin couplings with sub-mHz precision using zero- to ultralow-field nuclear magnetic resonance. J. Magn. Reson..

[47] Afach, S. Spintrum. https://git.afach.de/samerafach/Spintrum. (2018).

[48] Lloyd LS (2014). Hyperpolarisation through reversible interactions with parahydrogen. Catal. Sci. Technol..

