# Supplementary material for: Detection of pyridine derivatives by SABRE hyperpolarization at zero field
Source: Commun Chem. 2023 Jun 22;6:131. doi: 10.1038/s42004-023-00928-z (PMC10287679; doi:10.1038/s42004-023-00928-z)
Supplement: Supplementary file 1 — Supplemental Information [file 42004_2023_928_MOESM1_ESM.pdf]

Supplementary Information: Detection of Pyridine Derivatives by SABRE Hyperpolarization at Zero Field

Piotr Put,[1,*] Seyma Alcicek,[1,2,†] Oksana Bondar,[1,3] Łukasz Bodek,[1] Simon Ducket,[4] and Szymon Pustelny[1]

[1] *Institute of Physics, Faculty of Physics, Astronomy and Applied Computer Science, Jagiellonian University in Kraków, 30-348 Kraków, Poland*

[2] *Institute of Neuroradiology, University Hospital Frankfurt, Goethe University, Frankfurt, 60528, Germany.*

[3] *Department of Chemistry, Taras Shevchenko National University of Kyiv, 64 Volodymyrska St, 01601 Kyiv, Ukraine.*

[4] *Centre for Hyperpolarization in Magnetic Resonance (CHyM), University of York, Heslington, YO10 5NY, UK.*

SUPPLEMENTARY NOTE 1: BUBBLING SYSTEM AND EXPERIMENTAL SEQUENCE

The home-made bubbling system was used to purge NMR samples with nitrogen or parahydrogen gas. The schematics of the gas manifold are shown in Supplementary Figure 1a. The system had an inlet for N_2 to remove oxygen from the system and another inlet for pH_2 used for hyperpolarization. Both inlets could be closed from the system with manual valves (MVs). The pressure regulator reduced the nitrogen-gas pressure (PR) to a 4-5 bar level. The gas manifold had two short lines, one operated manually with MV and the other operated with the solenoid valve (SV). These lines allowed the gas to bypass the capillary submerged in an NMR sample, and hence equalized the pressure on both sides of the capillary. The outlet of the system could be cut off with SV, while the outflow of the gas was controlled with a needle valve (NV). To either purge the sample from oxygen or to initiate the SABRE process, the sample was first exposed to the gas by opening the gas cylinder with the short valves open (so that there was no pressure difference across the capillary). The manual short was then closed. To start bubbling in an experimental sequence, the SV-operated short was closed while the outlet was opened. To stop the bubbling prior to measurement, the outlet SV was closed while the short SV was opened to immediately equalize the pressure and eliminate bubbles from the sample. The sample itself was attached to the system via a 1/4 inch teflon tube stretched over the top of the 5-mm NMR tube. The capillary tube (0.012-inch inner diameter) that provided the gas for the bubbling was fully submerged in the liquid sample.

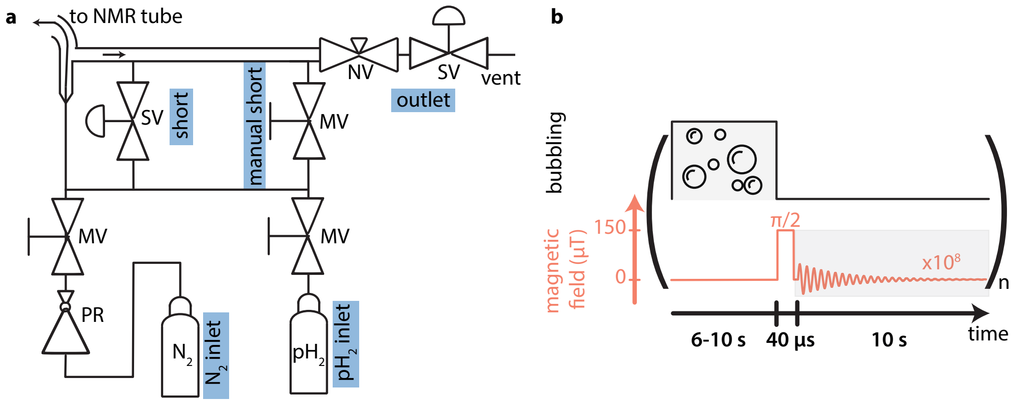

Supplementary Figure 1. **Experimental details of the system for zero-field SABRE-polarized NMR.** a) Schematics of the gas manifold used to bubble parahydrogen through the NMR sample. b) Experimental sequence used for zero-field experiments with SABRE hyperpolarization. The zero-field signal has been magnified about eight orders of magnitude to make it visible.

All experiments presented in this work followed an experimental sequence presented in Supplementary Fig 1b. Parahydrogen bubbling took place under zero-field conditions for 6-15 s. The bubbling was then stopped suddenly ($< 100\,\mathrm{ms}$) and a sharp DC pulse ($\sim 40\,\mu\mathrm{s}$ long) along the detection (z-)axis was applied. The length of the pulse

* put.piotr@gmail.com

† seyma.alcicek@kgu.de

corresponded to the $\pi/2$ rotation of protons, converting the singlet order created upon polarization to observable magnetization (for more details, see, e.g., Ref.[1]). The acquisition started as soon as the pulse was applied (the initial data, corrupted by the saturation of the magnetometers with the DC-field pulse, was removed from the analyzed signal). The sequence could be repeated many times to obtain signals at natural isotopic abundance.

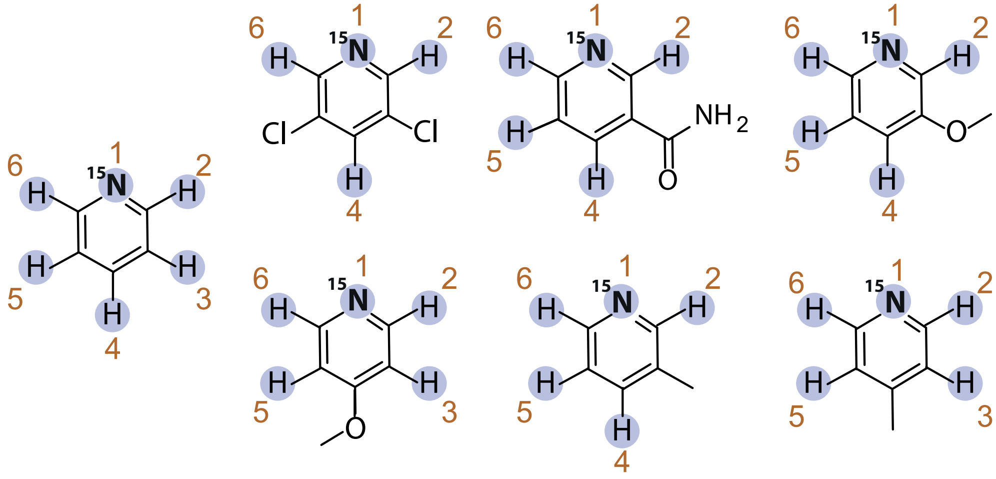

Supplementary Figure 2. **Chemical structures for ^{15}N-pyridine derivatives studied in this work.** The assigned numbers for ^{15}N and ^{1}H nuclei are used to show J-coupling constants in Supplementary Table 1, 2, 3, and 4.

Supplementary Table 1. ^{15}N-^{1}H and ^{1}H-^{1}H J-coupling constants of neat ^{15}N-pyridine and ^{15}N-pyridine in methanol solution[1] (See Supplementary Figure 2 for the nuclei in the molecular structure corresponding to each number).

J-coupling	Neat ^{15}N-pyridine (Hz)	^{15}N-pyridine in methanol (Hz)
J_{12}	-10.93	-10.14
J_{13}	-1.47	-1.56
J_{14}	0.27	0.18
J_{23}	4.88	4.87
J_{24}	1.83	1.85
J_{25}	0.97	1.01
J_{26}	-0.12	-0.15
J_{34}	7.62	7.65
J_{35}	1.38	1.35
J_{36}	0.97	1.01
J_{45}	7.62	7.65
J_{46}	1.83	1.85
J_{56}	4.88	4.87

Supplementary Table 2. ^{15}N-^{1}H and ^{1}H-^{1}H J-coupling constants of ^{15}N-nicotinamide and ^{15}N-3,5 dichloropyridine[2,3] (See Supplementary Figure 2 for the nuclei in the molecular structure corresponding to each number).

J-coupling	^{15}N-nicotinamide (Hz)	^{15}N-3,5 dichloropyridine (Hz)
J_{12}	-10	-10.6
J_{13}	-	-
J_{14}	-	-
J_{15}	-1.7	-
J_{16}	-10.3	-10.6
J_{23}	-	-
J_{24}	2.3	0.25
J_{25}	0.8	-
J_{26}	-	-
J_{34}	-	-
J_{35}	-	-
J_{36}	-	-
J_{45}	8.0	-
J_{46}	1.6	0.25
J_{56}	4.9	-

Supplementary Table 3. ^{15}N-^{1}H and ^{1}H-^{1}H J-coupling constants of ^{15}N-3-methoxypyridine and ^{15}N-4-methoxypyridine in methanol[4] (See Supplementary Figure 2 for the nuclei in the molecular structure corresponding to each number).

J-coupling	^{15}N-3-methoxypyridine (Hz)	^{15}N-4-methoxypyridine (Hz)
J_{12}	-10	-10.6
J_{13}	-	-1
J_{14}	-1.4	-
J_{15}	-	-1
J_{16}	-10	-10.6
J_{23}	-	5.75
J_{24}	3	-
J_{25}	0.7	0.6
J_{26}	-0.35	-0.2
J_{34}	-	-
J_{35}	-	2.6
J_{36}	-	0.6
J_{45}	8.65	-
J_{46}	1.4	-
J_{56}	4.75	5.75

Supplementary Table 4. ^{15}N-^{1}H and ^{1}H-^{1}H J-coupling constants of ^{15}N-3-methylpyridine and ^{15}N-4-methylpyridine[3] (See Supplementary Figure 2 for the nuclei in the molecular structure corresponding to each number).

J-coupling	^{15}N-3-methylpyridine (Hz)	^{15}N-4-methylpyridine (Hz)
J_{12}	-11.2	-10.1
J_{13}	-	-1.85
J_{14}	-0.4	-
J_{15}	-	-1.85
J_{16}	-11	-10.1
J_{23}	-	5.2
J_{24}	2.2	-
J_{25}	0.3	0.8
J_{26}	-0.35	0.4
J_{34}	0.7	0.7
J_{35}	-	1.7
J_{36}	-	0.8
J_{45}	7.8	0.7
J_{46}	2.2	-
J_{56}	4.4	5.2

[1] T. Theis, M. P. Ledbetter, G. Kervern, J. W. Blanchard, P. J. Ganssle, M. C. Butler, H. D. Shin, D. Budker, and A. Pines, Journal of the American Chemical Society **134**, 3987 (2012).

[2] R. V. Shchepin, D. A. Barskiy, D. M. Mikhaylov, and E. Y. Chekmenev, Bioconjugate Chemistry **27**, 878 (2016).

[3] W. Brügel, Zeitschrift für Elektrochemie, Berichte der Bunsengesellschaft für physikalische Chemie **66**, 159 (1962).

[4] U. Vögeli and W. von Philipsborn, Organic Magnetic Resonance **5**, 551 (1973).
