# Supplementary material for: Detection of pyridine derivatives by SABRE hyperpolarization at zero field
Source: Commun Chem. 2023 Jun 22;6:131. doi: 10.1038/s42004-023-00928-z (PMC10287679; doi:10.1038/s42004-023-00928-z)
Supplement: Supplementary file 2 — Description of Additional Supplementary Files [file 42004_2023_928_MOESM2_ESM.pdf]

Description of Additional Supplementary Files

File name: Supplementary Data 1

Description: Raw experimental and simulated NMR spectra

File name: Supplementary Data 2

Description: Zero-field NMR spectra simulation code based on the Spintrum library
